# Society of Obstetricians and Gynecologists Pakistan Guideline on Second Trimester Anomaly Scan Published in year 2022

**DOI:** 10.12669/pjms.38.7.6212

**Published:** 2022

**Authors:** Nishat Zohra, Shama Munim, Shamila Ijaz, Shehla Baqai, Haleema Yasmin, Razia Korejo, Lees CC

**Affiliations:** 1 Nishat Zohra, Isra University Hyderabad, Pakistan; 2 ShamaMunim, Jinnah Medical and Dental University, Karachi, Pakistan; 3 Shamila Ijaz, Allama Iqbal Medical College, Lahore, Pakistan; 4 Shehla Baqai, CMH Lahore Medical College, Lahore, Pakistan; 5 Haleema Yasmin, Jinnah Postgraduate Medical Center, Karachi, Pakistan; 6 Razia Korejo, Baharia University, Karachi, Pakistan; 7 Lees CC, Imperial College London, UK

**Keywords:** Second trimester, Anomaly scan, Maternal and fetal medicine guidelines

## Abstract

The Guideline on Second trimester anomaly scan has been prepared by the National Maternal Fetal Medicine guidelines committee, approved by the Society of Obstetricians and Gynecologists Pakistan. These guidelines are developed in 2022 and will be reviewed after two years.

The current document provides guidance about the importance of second trimester scan to practicing clinicians and sonologists. It will enable them to offer it timely offer to their patient this scan and refer them to the Fetal medicine specialist when indicated. It is unique as the document is modified according to local needs. The Guidelines are developed in 2022 and will be reviewed after two years.

## INTRODUCTION

Congenital anomalies are a major cause of perinatal morbidity and mortality. Timely screening and detection of these anomalies by a non-invasive and cost-effective method of sonography can be done in majority of such cases. These guidelines are prepared by a group of experts from Pakistan to develop a consensus-based recommendations of performing second trimester scan that can be adopted throughout the country.

Second trimester anomaly scan is performed to detect structural anomalies and identify markers for chromosomal anomalies. At this time placental localization and liquor volume are also assessed and dating of the pregnancy is done for those attending late.^1^

It is best performed between 18 – 22 weeks of Gestation, In Pakistan we recommend anomaly scan earlier by 20 weeks for timely detection of anomalies and decision for continuation of pregnancy.^1^ (level of evidence 111). It is offered to all pregnant women specially those having risk of structural and chromosomal anomalies. It is performed by Radiologist, Sonologist and Maternal Fetal Medicine experts. However , we recommend that scan should be performed by a certified operator / Fetal Medicine specialist having expertise in identifying the fetal anomalies and providing appropriate counseling.^2^

### 1.1: Women more at risk for having structural Abnormalities:^2^

1. Advanced maternal age (>35 years)
2. Previous fetus or child with chromosomal and structural abnormality.
3. Previous child with Genetic Syndrome.
4. First Trimester scan showing Nuchal translucency 3 mm or more.
5. Abnormal maternal serum markers in 1^st^ trimester.
6. Fetal growth restriction in the current Pregnancy.
7. Pregestational diabetes or gestational diabetes diagnosed before24 weeks.
8. Multiple or higher order pregnancy.
9. Teratogen exposure and maternal drug use.
10. Congenital fetal infections.
11. Oligo and Polyhydramnios

### 1.2: Common Ultrasound markers associated with a Chromosomal abnormality are:

**Table:** 

	DON’T MISS	YOU MIGHT MISS	YOU WILL MISS
Brain andSkull	Ventriculomegaly,	Partial agenesis of Corpus	Lissencephaly White matter lesions Infarcts
	Dandy Walker Malformation,	Callosum	
	Arnold Chiarri 11 Malformation	Vascular malformations	
	Agenesis of Corpus Callosum	Destructive lesions
Face	Cleft Lip	Superficial cleft lip	Isolated cleft palate
	Micraganthia	Mild Microganthia	
	Large Masses	Ear abnormaolities	
Neck	Nuchal Edema	Small neck mass	Thyroid anomalies
	Cystic Hygroma	
	Large neck mass		Laryngeal obstruction
Chest	Diaphragmatic hernia	Small chest masses	Diaphragmatic eventration
	Large chest mass		
	Pleural effusion		
Heart	Ventricular disproportion	Smaller VSD Aortic coarctation	ASD Anomaloud pulmonary venous drainage
	Univentricular heart		
	Pentalogy of Cantrell		
	Large VSD		
	Arrythmia		
	Tetralogy of Fallot		
	Transposition of great vessels		
	Truncus arteriosus		
Abdomen	Gastroschisis	Choledochal cyst Esophageal atresia	Intestinal atresia Anal atresia
	Omphalocele		
	Ascites		
Genitourinary	Single umbilical artery	Unilateral renal agenesis Ectopic kidney Ambiguous genitalia	hypospadias
	Renal agenesis		
	Multicystic kidneys		
	Hydronephrosis		
	Megacystis		
	Bladder exstrophy		
Spine	Spina bifida Sacrococcygealteratoma	Hemivertebra Spinabifidaocculta	Tethered cord
Extremities	Limb reduction defects	Polydactyly Syndactyly Soft tissue abnormalities	Mild skeletal dysplasia
	Micromelia (short lims)		
	Fractures and bowing		
	Joint contractures		
	Absent thumb

1. Nuchal Edema.
2. Ventriculomegaly.
3. Cardiac defects.
4. Echogenic Bowel.
5. Short Femur/Humerus.
6. Duodenal Atresia.
7. Renal Pelvic Dilatation.

### 1.3: Which Abnormalities are detected by the Ultrasound?

All fetal conditions are not diagnosed by ultrasound. They can be categorized into those that are diagnosed most of the time and should not be missed on ultrasound. While some conditions can be missed on the scan and some are those that are not diagnosed by ultrasound.^3^

### 1.4: How to report Anomaly scan:

- Patients Biodata:
- Name, Husband name, Age, Parity,
- Family history of congenital anomalies.
- Last Menstural period (LMP) and Expected date of delivery (EDD)
- Indication of Anomaly scan:
- Presentation and lie of fetus
- Placental position. Any placental abnormality.

### 1.4.1: Biometric measurements include:

- Biparietal diameter, Occipito frontal diameter (OFD)
- Head circumference.
- Femur and Humerus.
- Ventricular atrium.
- Transverse Cerebellar diameter (TCD), Cisterna magna.
- Abdominal circumference
- Amniotic fluid index (AFI)& Estimated fetal weight (EFW)
- Doppler where applicable

### 1.4.2: Anatomical survey: 4

**•Head**: Intact cranium, shape
**•Brain** - Cavum septum pellucidum, Midline falx, Thalami, Cerebral ventricles, Choroid plexus, Cerebellum and Cisterna magna.
**•Face -** Both orbits, Median facial profile, Mouth, Upper lip.
**•Neck –**Any mass, cystic hygroma
**•Chest-** lungs, pleural effusion, Diaphragm
**•Abdomen** - Abdominal organs, their situs, Stomach and intestines, Kidneys and urinary bladder. Ascites, abdominal mass and abdominal wall defects.
**Heart:** Cardiac activity, Four-chamber view of the heart, Outflow tracts andthe three-vessel and tracheal view where possible.
Spine and Limbs.

### 2.0: Invasive Procedures:

Before performing the invasive procedure, couple is counseled regarding the procedure, its risks, benefits and outcomes. Informed decision about undergoing these tests is made by the couple. A prior written consent is obtained mentioning the risk of miscarriage in these procedures (0. 22% for CVS and 0.11 % for amniocentesis).^5^

Invasive Procedures like CVS and Amniocentesis need to be performed by a certified operator.

### 2.1: Indications for invasive procedures:

- History of previous baby with Chromosomal abnormalities.
- Presence of sonographic marker or structural abnormality is the commonest indication for invasive testing.
- Genetic Syndromes or single Gene disorders in family.

### 3:0: Indication of Fetal echocardiography^6^:

- Women above 35 years of age.
- Abnormal four chamber view or three vessel view or Fetal arrhythmia detected by routine ultrasound.
- Raised Nuchal Translucency (>3 mm).
- Fetal Chromosomal abnormality.
- Family history of congenital heart disease,
- Maternal diabetes, Thyroid disorder or systemic lupus erythematosus.
- Fetal exposure to a teratogen or drugs like Ace inhibitors, NSAIDS, Anti epileptics
- Multiple Pregnancy in particular Monochrionic twins.

Repeat ultrasound scan is offered if first scan is not complete due to maternal obesity, uterine Fibroids or sub optimal position of baby.^6^

### 4.0: Who should perform mid trimester scan?

- Operator should be Certified and trained in Performance and interpretation of a detailed fetal scan.
- Operator has special expertise in the identification and diagnosis of fetal anomalies.

### 5.0: What type of Equipment is needed? Equipment should have following capabilities:

- Real time, grey scale ultrasound capabilities
- Curvilinear probe
- Color Doppler
- Transabdominal ultrasound transducers (3 – 5 MHz range)
- Freeze frame capabilities
- Electronic calipers
- Capacity to print/store images
- Regular service record

### 6.0: Is Prenatal Ultrasonography Safe?

- Safe for clinical practice.
- Fetal exposure times should be minimized,
- Use lowest possible power output.

### Recommendations:

- Every pregnant patient should be offered second trimester anomaly scan.
- Detailed anatomic survey is mandatory at 18-20 weeks scan
- Liquor and Placental abnormalities are to be excluded at this time
- If any anomaly is suspected or detected patient is referred to Fetal Medicine specialist
- for further management.
- All sonographers should have proper training for doing 2^nd^ trimester fetal ultrasound.

### Author`s Contribution:

**NZ:** Designed and prepared the manuscript

**SM:** Edited the manuscript

**SE:** Data collection

**SB:** Critically reviewed the manuscript

**HY and RK:** Conceived the idea of making SOGP MFM guidelines according to needs of Pakistan.

Lees CC UK Based expert on the subject has reviewed these guidelines.

### Note

Guidelines are prepared by Prof. Nishat Zohra with input from members of Guideline committee including Prof. Shehla Baqai, Prof. Halima Yasmin and Prof. Shamila Ijaz in consultation and review by Chair of guideline committee Prof. Shama Munim. Nishat Zohra takes the responsibility and is accountable for the content of article.
